# Evaluation of Myosin Heavy Chain Isoforms in Biopsied *Longissimus Thoracis* Muscle for Estimation of Meat Quality Traits in Live Pigs

**DOI:** 10.3390/ani10010009

**Published:** 2019-12-19

**Authors:** Min Young Park, Youn-Chul Ryu, Chung-Nam Kim, Kyung-Bo Ko, Jun-Mo Kim

**Affiliations:** 1 Department of Animal Science and Technology, College of Biotechnology and Natural Resources, Chung-Ang University, Ansung-si Gyeonggi-do 17546, Korea; bennjerrry@hotmail.com; 2 Division of Biotechnology, SARI, Jeju National University, Jeju-do 63243, Korea; ycryu@jejunu.ac.kr (Y.-C.R.); kch1118@nate.com (C.-N.K.); kkb5240@jejunu.ac.kr (K.-B.K.)

**Keywords:** myosin heavy chain, *longissimus thoracis*, biopsy, meat quality, pigs

## Abstract

**Simple Summary:**

Pork quality has become an important parameter in the industry. Traditional pork quality was assessed postmortem. It is considered that the determination of meat quality in live pigs is beneficial in order to obtain better pork quality and to reduce cost in production. In the present study, myosin heavy chain (MHC) isoforms in both of the pre- and postmortem *longissimus thoracis* muscle were evaluated as novel parameters for meat quality estimation in pork by correlation and clustering analysis. MHC isoforms in live pigs could be applied in a practical and useful method to predict meat quality in pork.

**Abstract:**

Estimating meat quality prior to slaughter will be beneficial for the rapid identification of specific traits or poor quality pork compared to a conventional assessment at postmortem. In this study, we identified and quantified myosin heavy chain (MHC) isoforms from a biopsied *longissimus thoracis* muscle of pigs, and determined their correlation with postmortem muscle fiber characteristics and meat quality. MHC slow and fast isoforms proportions from biopsied samples correlated with postmortem percentage of type I and type IIB muscle fibers, respectively (*p* < 0.05). The percentage of the biopsied MHC slow isoform showed a positive correlation with pH at 45 min postmortem, and negative correlations with filter-paper fluid uptake and drip loss in pork (*p* < 0.05). Furthermore, clustering the pigs into three groups based on the biopsied MHC isoform proportions was not only significantly associated with muscle fiber number and proportions of muscle fiber area, but also correlated with pH at 45 min postmortem and the National Pork Producers Council color score (*p* < 0.05). Collectively, our findings indicate that the biopsied MHC isoforms serve as parameter for estimating meat quality, with the association between the higher proportion of MHC slow isoforms and pH at 45 min postmortem in particular being indicative of better pork quality.

## 1. Introduction

The measurement of meat quality has become an important criterion for establishing standards for pork production and consumption. Conventionally, meat quality is determined in the postmortem *longissimus thoracis* (LT) muscle; however, as an alternative, predicting meat quality in live animals can have several benefits. Estimation of meat quality from live animals can, for example, confirm genetic analysis by facilitating the identification of pigs containing specific genetic traits within a shorter period of rearing until slaughter [1], and can also be used to avoid the production of pigs prone to pale, soft, and exudative (PSE) meat [2].

The criteria used for assessing meat quality include pH, meat color, water-holding capacity, cooking loss, and tenderness. Various measurement methods have been introduced for the improvement of these traits, including the use of different breeds and adopting different feeding and pork management strategies [3]. To a large extent, the aforementioned criteria depend upon skeletal muscle fiber characteristics (MFCs), including muscle fiber number and size and fiber-type composition [4,5]. Skeletal muscle is composed of muscle fibers that can be classified as either type I (slow-twitch) or type II (fast-twitch), based on myosin heavy chain (MHC) isoform expression [6]. There are three major types of MHC isoforms, namely, I, IIA, and IIB. Type I fibers mainly consist of MHC I isoforms and contract slowly, as ATP is generated via the oxidative pathway. In contrast, type II fibers are composed of MHC IIA, IIX, and IIB isoforms, which utilize the glycolytic pathway for ATP production [7]. The biochemical properties of muscle fibers comprising different MHC isoforms affect postmortem carcass metabolism. Consequently, meat quality has consistently been shown to be correlated with fiber-type composition, particularly compared with other MFCs [8].

Previous studies have reported that MHC isoforms are strongly correlated with the postmortem metabolic rate and consequently meat quality traits. For example, it has been found that the rate and extent of postmortem pH decline are greater in meat with a higher proportion of fast-twitch glycolytic fibers, which in the porcine *longissimus* muscle consist predominantly of MHC fast isoforms [4,9,10], ultimately leading to PSE meat. In addition, it has been shown that the MHC slow isoform is positively correlated with pH at 24 h postmortem and negatively correlated with drip loss [11]. Lametsch et al. concerned postmortem MHC degradation [12]; however, it is still a valid criterion because conventionally carcass is stored at low temperature where MHC degradation results in a lesser extent [13,14]. Moreover, our previous study suggested MHCs in live pigs as a proper standard for estimating meat quality [15]. 

Therefore, in the present study, we measured the slow and fast isoforms of MHC in biopsied samples of the *Longissimus thoracis* muscle obtained from live pigs and examined correlations between the MHC isoforms in these samples and postmortem MFC, as well as meat quality.

## 2. Materials and Methods

### 2.1. Animals and Muscle Samples

All the experimental procedures performed in this study were approved by the Animal Ethics Committee of Jeju National University (Approval No. 2016–0057 Jeju, Korea). A total of 668 Jeju black commercial pigs (146 castrated males, 92 intact males, and 386 females) were used in the study, as described in our previous study [15]. All pigs were raised under controlled conditions and were provided with water and diet ad libitum. Biopsy samples for MHC isoform analyses were collected from the live pigs at the end of their growing phase (106.9 ± 7.9 days) and samples used for the determination of postmortem muscle fiber characteristics were collected from the carcasses of slaughtered pigs at 45 min postmortem (213.5 ± 17.5 days). At both time points, muscle samples for muscle fiber characteristic determinations were collected from the *longissimus thoracis* located around the 8th to 9th thoracic vertebrae with a lateral distance of 6.5 cm from the midline using a disposable biopsy needle of 12 cm length and 1.8 mm diameter (Guideneedle; Truguide, Bard, Tempe, AZ, USA). Collected samples were immediately frozen in liquid nitrogen and stored at −80 °C until analysis. For meat quality assessment, pH was measured in carcasses at 45 min postmortem, and other parameters were measured from the samples obtained after chilling at 4 °C for 24 h postmortem. Samples for meat quality analysis were collected in the LT area from the 10th to 13th thoracic vertebrae.

### 2.2. Determination of MHC Isoforms and Muscle Fiber Characteristics in Biopsied LT Samples

The MHC isoforms of biopsied LT samples were identified and quantified by gel electrophoresis, according to the previous study [16]. Briefly, MHC isoforms were analyzed by SDS-PAGE for separation of slow and fast isoforms. After visualizing the bands with Coomasie blue staining, we quantified the band density using the Kodak 1D image analysis software (Eastman Kodak Co., Rochester, NY, USA). The postmortem samples were examined for muscle fiber types I, IIA, and IIB using myofibrillar adenosine triphosphatase staining methods and classified according to nomenclature of Brooke and Kaiser [7]. Histochemically stained muscle sections were analyzed using an optical image analysis program (Image-Pro plus; Media Cybernetics, L.P., Rockville, MD, USA). The cross-sectional area of muscle fibers was expressed as the ratio of muscle fiber area to total muscle fiber count. The proportion of muscle fiber area accounts for the ratio of total cross-sectional area (CSA) of each fiber type to total measured fiber area. The proportion of muscle fiber number was obtained from the ratio between each fiber type number to total fiber number.

### 2.3. Measurement of Meat Quality

The muscle pH was measured at 45 min postmortem using a spear-type portable pH meter (MH-17mX; Toadkk, Tokyo, Japan) after calibration with standard solutions at pH 4 and pH 7 and automatic temperature compensation. After carcasses were chilled at 4 °C for 24 h, cuts of fresh meat samples were collected and used to determine meat quality traits, including meat color, filter-paper fluid uptake (FFU), drip loss, and cooking loss. For meat color assessment, fresh meat samples were bloomed by exposing to air at 4 °C for 30 min and measurements were performed using a Minolta chromameter (CR-300; Minolta Camera Co., Tokyo, Japan). The values for color were expressed in terms of three color coordinates (L*, a*, b*) from triplicate measurements. For FFU, meat samples were exposed to air for 15 min, and filter paper of known weight was placed in contact with the meat samples for 2 s. FFU values were determined from changes in filter paper weight. Drip loss was calculated by weight difference between the initial and final weight of samples after suspending a sample in an inflated bag at 4 °C for 48 h, and expressed as a percentage of sample weight change. For cooking loss, meat samples were sealed in a plastic bag and cooked by placing at 80 °C. When the internal temperature reached 75 °C, measured by using a thermometer with probe (TES-1300, TES Electrical Electronic Co., Taipei, Taiwan), the samples were maintained at this temperature for 10 min until the completion of cooking. Cooking loss was calculated as the change in sample weight between before and after cooking. Additionally, after exposure to air at 4 °C for 30 min, 12 panellist evaluated fresh pork for color (1 to 5 from pale to dark) and marbling (1 to 10 from low to dark) scores according to the National Pork Producers Council (NPPC) standards (2000).

### 2.4. Statistical Analysis

Analyses of correlations and associations between variables were performed using the SAS 9.2 statistical software (SAS Institute, Inc., Cary, NC, USA). For preliminary clustering analysis, the pigs were clustered based on three variables of MHC isoforms using the FASTCLUS procedure in SAS. After the pigs were hierarchically categorized into 10 pre-clusters (Figure S1), semi-partial R-squared values were used to determine branch distances between each pre-cluster level. Thereafter, the 10 pre-clusters were further categorized into three clusters using Ward’s minimum-variance method [17]. Finally, principal component analysis (PCA) was performed to determine correlations between the biopsied MHC isoform ratio and the postmortem MFCs and meat quality traits. The general linear model used to analyze the associations between the clusters and the measured traits was as follows:

*y_ijklm_* = *μ* + *C_i_* + *S_j_* + *D_k_* + *P_l_* + *e_ijklm_*, (1)

where *y_ijklm_* is the observation of the traits, *μ* is the general mean, *C_i_* is the fixed effect of cluster *i*, *S_j_* is the fixed effect of sex *j*, *D_k_* is a covariation for sampling age in biopsy or slaughter *k*, *P_l_* is the fixed effect of sire, and *e_ijklm_* is the random error. The results were presented as least-square means and standard errors, and significant differences between the cluster groups were determined by the probability difference (PDIFF) option using SAS 9.2 (SAS Institute, Inc., Cary, NC, USA).

## 3. Results and Discussion

### 3.1. Correlation between MHC Isoforms in Biopsied LT Muscle and Muscle Fiber Characteristics and Meat Quality in Postmortem LT Muscle

MHC isoforms in biopsied LT, and MFC and meat quality in postmortem LT, were measured from a total of 668 pigs. Summary statistics are presented in Table S1. Consistent with the findings of previous studies, our results showed that MHC fast isoforms in biopsied samples and type IIB fibers at postmortem were the predominant fiber types in LT [10]. 

We then determined correlation coefficients between the percentage of the biopsied MHC isoforms from the biopsied LT muscle and the muscle fiber traits of postmortem LT muscle (Table 1). As previously reported, type I fibers mainly comprise slow isoform MHC [6], and we detected a positive correlation between the biopsied MHC slow isoform and the proportion of postmortem type I muscle fiber area (r = 0.513, *p* < 0.001), as well as the number of type I muscle fibers (r = 0.580, *p* < 0.001). In contrast, a negative correlation was observed between the biopsied MHC slow isoform and postmortem type IIB muscle fiber area (r = −4.440, *p* < 0.001) and the number of type IIB muscle fibers (r = −0.496, *p* < 0.001). Both the percentage of MHC fast isoform and the ratio of MHC fast to slow isoform of the biopsied sample were positively correlated with the proportion of area and fiber number of type IIB fiber at postmortem, whereas we detected negative correlations with the same variables in postmortem type I fibers. Overall, the MHC isoforms in the biopsy samples showed relatively higher coefficients for correlations with the proportion of muscle fiber area and muscle fiber number at postmortem than for correlations with other variables. These results are consistent with the findings of our previous study in which we showed that biopsied and postmortem muscle fiber characteristics were significantly correlated [15]. The coefficients of correlations between MHC isoforms in the biopsied LT and the cross-sectional area of postmortem muscle fiber were relatively lower, which might be attributable to large variations in size of the skeletal muscle of individual pigs sampled at different dynamic stages of muscle fiber. 

We also determined the correlations between the percentage of each MHC isoform in the biopsy samples and postmortem meat quality traits (Table 1). Compared with the other assessed variables, MHC slow isoforms showed the strongest correlation with pH at 45 min postmortem (r = 0.521, *p* < 0.001). Postmortem pH is largely affected by glycogen and lactate contents, which are stored in different amounts in different muscle fiber types. Furthermore, it has been suggested that muscle fibers with low glycogen and lactate content and higher postmortem pH are composed of a higher proportion of type I fibers [18]. Although we did not determine lactate contents in the present study, our results partly correspond with the correlation between the proportions of MHC slow isoform in biopsied samples and a higher postmortem pH. FFU and drip loss are indices of the water-holding capacity of meat. In the present study, these criteria were shown to be negatively correlated with MHC slow isoform, albeit with low r values (r = −0.138 and r = −0.145, *p* < 0.001). As previously suggested by Valin et al. [19], the biopsied muscle could be used for meat quality assessment in lamb due to the minimal difference in muscle fiber types in biopsy and postmortem material, which is in line with the findings of the present study that highlight the value of biopsied meat analysis for the estimation of postmortem meat quality. In summary, our analysis of the association between MHC isoforms from the biopsied LT muscle and meat quality indicated that the proportion of MHC slow isoform in the biopsied muscle is positively correlated with the pH of muscle samples at 45 min postmortem, and negatively correlated with L*, FFU, and drip loss.

### 3.2. Effects of MHC Isoform Clustering in Estimating Postmortem Meat Quality

On the basis of the characteristics of MHC isoforms in the biopsied LT, all the pigs used in the present study were pre-clustered into 10 groups (Figure S1 and Table S2) [20], and finally clustered into three groups (Table 2). Classification into these three clusters was based on the percentage of MHC slow isoform, with cluster 1 having the smallest percentage and cluster 3 having the largest. Correspondingly, the percentage of MHC fast isoform was highest in cluster 1 and lowest in cluster 3 (*p* < 0.001). Overall, cluster 1 was characterized by the highest ratio of MHC fast to slow isoform, whereas cluster 3 had the smallest ratio (*p* < 0.001). Evaluation of clustering was performed by estimating the canonical coefficients of the three clusters (Figure S2), and as depicted in this figure, individual pigs in the same cluster were aligned together with represented different colors. Having clustered the pigs, we then determined the effects of MHC isoform cluster on muscle fiber characteristics (Table 3). Compared with the results for MHC isoforms prior to clustering, we noted differences in the significance of variables, including total muscle fiber number and the density and cross-sectional area of muscle fibers. However, the overall trend in the relationship between the MHC clusters and proportion of muscle fiber area and fiber number (*p* < 0.001) were similar. As cluster 3 had the largest percentage of the MHC slow isoform, this cluster was also associated with the highest percentages of type I muscle fiber area and fiber number, and the lowest percentages of type IIB fibers.

**Figure 1:**
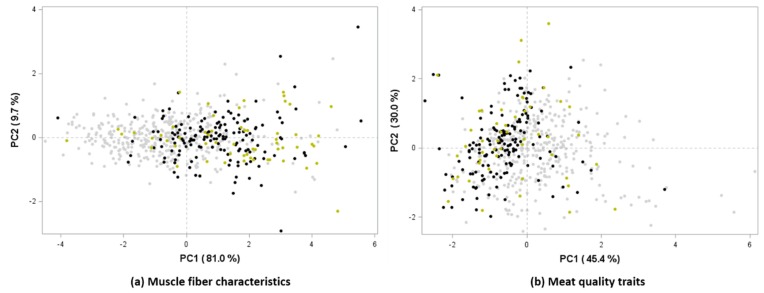
Principal component analysis (PCA) based on the significantly different myosin heavy chain isoform clusters. (**a**) Muscle fiber characteristics. PCA using the significant muscle fiber characteristics (*p* < 0.01): proportions of muscle fiber type I and IIB areas and numbers. (**b**) Meat quality traits. PCA using the significant meat quality traits (*p* < 0.01): pH_45min_, lightness, and drip loss. Individual eigenvalues of the first two major principal components (PC1 and PC2) are presented by the colored scatter plots: gray, cluster 1; black, cluster 2; yellow, cluster 3.

We also determined the effects of the biopsied MHC isoform clusters on meat quality estimation (Table 4). Consistent with the lowest ratio of MHC slow to fast isoforms in cluster 1, the pH at 45 min postmortem was significantly lower in this cluster compared with that in clusters 2 and 3 (*p* < 0.001). These results are in line with the findings of Yang et al. [11], who demonstrated that a higher slow MHC isoform content is correlated with pH at 24 h postmortem. Additionally, NPPC color, which initially did not show a significant correlation with MHC isoforms, exhibited significant differences among clusters, with cluster 1 showing lowest and cluster 3 the highest values. However, clustering had no significant affect on L* and b* values among meat color variables, nor on drip loss or cooking loss. It is considered that variations in meat quality traits were relatively greater in cluster 3, which affected the overall average value. Finally, on the basis of PCA, we validated the clustering of MHC isoforms in the biopsied muscle for its relationship with muscle fiber characteristics and meat quality (Figure 1). For muscle fiber characteristics, we found that PC1 accounts for 81% of the variation, whereas PC2 accounts for 9.7% (Figure 1a), with each of the three clusters being scattered along the PC 1 axis. For meat quality assessment, PC1 and PC2 explained 45.4% and 30% of the variation, respectively (Figure 1b), with clusters 2 and 3 being separated from cluster 1 along the PC1 axis. 

In summary, we characterized MHC isoforms in the biopsied LT and observed significant correlations with postmortem MFC and meat quality. Correlations between the proportion of the biopsied MHC isoforms and postmortem MFC were significant, as relatively high r values were obtained for the relationships between the proportion MHC isoforms and muscle fiber number and area. Regarding its relationship with meat quality, the percentage of MHC slow isoform in the biopsied muscle showed a positive correlation with pH at 45 min postmortem, and was negatively correlated with the ratio of the MHC fast to slow isoforms and drip loss. Overall, these findings indicate that the MHC isoforms measured in the biopsied LT are highly correlated with the MFC and meat quality determined in postmortem samples. When we applied clustering analysis based on the characteristics of MHC isoforms, we found that the results obtained, after clustering pigs into three groups based on the proportions and ratios of MHC isoforms, revealed relationships with MFC and meat quality comparable to those obtained in our analyses of individual pigs.

Previously, several strategies have been introduced for the estimation of meat quality in live pigs. However, although each of these methods can be readily applied in the field, they show certain limitations with regards to estimating meat quality. Detection of halothane gene variation in live pigs has been used as a basis for the elimination of those carcasses with the possibility of PSE meat, but is not recommended for postmortem meat quality assessment [2]. Measuring the rectal temperature of pigs is common and has shown a correlation with pH, although it takes time [21,22]. Ultrasound techniques have also been applied to predict intramuscular fat (IMF) in vivo; however, they are less applicable due to a large variation during measurement [23]. Another recent research suggested using a portable hyperspectral scanner to monitor pH of meat [24]. However, despite of direct associations of IMF with sensory quality traits, it has yet been widely implemented in meat quality assessment because of individual variations in IMF [25]. MFC traits in a biopsied LT sample have also been suggested to have potential utility for the estimation of meat quality [15], and the identification of muscle fiber type can also be undertaken using immunohistochemical techniques. However, the histological analyses involved in both these approaches are time-consuming and labor-intensive. Determination of MHC isoforms is a comparatively simple and rapid method for application and requires less sampling from pigs. Taken together, the results of the present study lead us to suggest that variations in MHC isoforms in the biopsied LT muscle are closely related to postmortem MFCs and conventional meat quality traits. We accordingly conclude that MHC isoforms determined from live pigs represent a reliable and readily measured criterion for estimating the ultimate quality of pig meat after slaughter. For more precise estimations of meat quality, however, further studies are necessary for the standardization of sampling point, age, and growth stage to enable a routine application of this approach.

## 4. Conclusions

In this study, MHC isoforms were analyzed in the biopsied porcine longissimus thorasis (LT) muscle and their correlations with postmortem muscle fiber characteristics and meat quality were determined. Our results showed that slow and fast MHC isoforms in the biopsied muscle were positively correlated with the postmortem type I and type IIB muscle fiber characteristics, respectively. Moreover, the biopsied MHC isoforms showed significant correlations with meat quality criteria including pH and water-holding capacity. Consistently, clustering analysis according to the MHC isoforms in the biopsied LT muscle exhibited to relate with postmortem muscle fiber type composition and pH in meat quality. Therefore, our results suggest that biopsied MHC isoforms can be considered as indirect parameters for assessment of meat quality in pork. 

## Figures and Tables

**Table 1:** Correlation coefficients (*r*) of the biopsied myosin heavy chain (MHC) isoforms between postmortem muscle fiber characteristics and meat quality traits.

Variables	MHC Isoforms at Biopsy ^1^		
	Slow Isoform (%)	Fast Isoform (%)	Fast/Slow Ratio
Muscle fiber characteristics			
Total muscle fiber number	0.087	−0.087	−0.099 *
The density of muscle fibers	0.149 ***	−0.149 ***	−0.149 ***
Cross sectional area of muscle fiber			
Mean area	−0.144 ***	0.144 ***	0.128 **
Type I area	−0.202 ***	0.202 ***	0.241 ***
Type IIA area	−0.105 *	0.105 *	0.102 *
Type IIB area	−0.093 *	0.093 *	0.075
Proportion of muscle fiber area			
Type I	0.513 ***	−0.513 ***	−0.546 ***
Type IIA	0.006	−0.006	0.051
Type IIB	−0.440 ***	0.440 ***	0.438 ***
Proportion of muscle fiber number			
Type I	0.580 ***	−0.580 ***	−0.622 ***
Type IIA	0.005	−0.005	0.052
Type IIB	−0.496 ***	0.496 ***	0.496 ***
Meat quality traits			
pH_45min_	0.521 ***	−0.521 ***	−0.581 ***
L*	−0.091 *	0.091 *	0.092 *
a*	−0.002	0.002	0.009
b*	−0.056	0.056	0.049
FFU	−0.138 **	0.138 **	0.112 **
Drip loss_24h_	−0.145 ***	0.145 ***	0.197 ***
Cooking loss	−0.080	0.080	0.038
NPPC color	0.043	−0.043	−0.064
NPPC marbling	0.023	−0.023	−0.022

* *p <* 0.05; ** *p <* 0.01; *** *p <* 0.001.

**Table 2:** Identifications of myosin heavy chain (MHC) isoform cluster groups from the biopsied *longissimus thoracis* muscle by comparisons in pigs.

Variables	MHC Isoform Clusters			*p*
	Cluster 1 (n = 396)	Cluster 2 (n = 169)	Cluster 3 (n = 103)	
MHC isoform characteristics at biopsy				
MHC Slow isoform (%)	9.06 ± 0.34	25.12 ± 0.40	39.48 ± 0.49	***
MHC Fast isoform (%)	90.94 ± 0.34	74.88 ± 0.40	60.52 ± 0.49	***
MHC Fast/Slow ratio	12.12 ± 0.38	3.61 ± 0.46	1.86 ± 0.56	***

Values are presented as mean ± standard error. *** *p <* 0.001.

**Table 3:** Effects of the biopsied myosin heavy chain (MHC) isoform clusters on muscle fiber characteristics in the *longissimus thoracis* muscle.

Variables	MHC Isoform Clusters			*p*
	Cluster 1 (n = 396)	Cluster 2 (n = 169)	Cluster 3 (n = 103)	
Muscle fiber characteristics				
Total muscle fiber number (×10^3^)	1098.0 ± 23.8 ^a^	1063.0 ± 30.4 ^a,b^	983.0 ± 56.6 ^b^	NS
The density of muscle fibers (/mm^2^)	252.8 ± 3.8	247.9 ± 4.8	241.4 ± 7.2	NS
Cross sectional area of muscle fiber (mm^2^)				
Mean area	4137.0 ± 64.3	4227.0 ± 83.1	4292.0 ± 121.0	NS
Type I area	3353.0 ± 63.0	3318.0 ± 79.5	3362.0 ± 118.5	NS
Type IIA area	2581.0 ± 59.6 ^b^	2756.0 ± 75.2 ^a^	2777.0 ± 112.1 ^a,b^	*
Type IIB area	4411.0 ± 75.1	4543.0 ± 94.8	4652.0 ± 141.3	NS
Proportion of muscle fiber area (%)				
Type I	8.8 ± 0.2 ^c^	10.8 ± 0.3 ^b^	11.8 ± 0.4 ^a^	***
Type IIA	4.8 ± 0.2	5.1 ± 0.2	5.0 ± 0.3	NS
Type IIB	86.4 ± 0.3 ^a^	84.2 ± 0.4 ^b^	83.2 ± 0.5 ^b^	***
Proportion of muscle fiber number (%)				
Type I	10.9 ± 0.3 ^c^	13.7 ± 0.3 ^b^	15.0 ± 0.5 ^a^	***
Type IIA	7.8 ± 0.2	7.8 ± 0.3	7.8 ± 0.5	NS
Type IIB	81.4 ± 0.3 ^a^	78.5 ± 0.4 ^b^	77.2 ± 0.6 ^c^	***

^a,b,c^ Means within the same row with different superscripts significantly differ. * *p <* 0.05; *** *p <* 0.001.

**Table 4:** Effects of the myosin heavy chain (MHC) isoform clusters at biopsy on meat quality traits.

Variables	MHC Isoform Clusters			*p*
	Cluster 1 (n = 396)	Cluster 2 (n = 169)	Cluster 3 (n = 103)	
Meat quality traits				
pH_45min_	6.31 ± 0.02 ^b^	6.56 ± 0.03 ^a^	6.57 ± 0.04 ^a^	***
L *	46.41 ± 0.25 ^a^	45.47 ± 0.29 ^b^	46.19 ± 0.48 ^a,b^	**
a *	6.62 ± 0.10	6.69 ± 0.12	6.75 ± 0.19	NS
b *	2.13 ± 0.07 ^a^	1.93 ± 0.08 ^b^	2.08 ± 0.13 ^a,b^	NS
FFU	25.91 ± 2.44	20.38 ± 2.93	18.52 ± 4.98	NS
Drip loss_24h_	1.08 ± 0.08 ^a^	0.80 ± 0.09 ^b^	1.00 ± 0.15 ^a,b^	**
Cooking loss	20.83 ± 0.39 ^a^	19.74 ± 0.46 ^b^	20.32 ± 0.75 ^a,b^	NS
NPPC color	2.45 ± 0.06 ^b^	2.54 ± 0.07 ^a,b^	2.68 ± 0.11 ^a^	NS
NPPC marbling	1.57 ± 0.05	1.52 ± 0.06	1.60 ± 0.10	NS

^a,b,c^ Means within the same row with different superscripts significantly differ. ** *p <* 0.01; *** *p <* 0.001.

## Supplementary Materials

The following are available online at https://www.mdpi.com/2076-2615/10/1/9/s1, Figure S1: Cluster analysis dendrogram, Figure S2: Grouped-scatter plot by two dimensional canonical coefficients for three clusters, Table S1: Summary statistics for measured traits, Table S2: Description of cluster size by the two-step clustering analysis in pigs based on the biopsied MHC isoforms from the *M. longissimus thoracis*.

## Abbreviations

MHC: myosin heavy chain; PSE: pale, soft, and exudative; MFC: muscle fiber characteristics; IMF: intramuscular fat; LT: *longissimus thoracis*.
