# Supplementary material for: Evaluation of Myosin Heavy Chain Isoforms in Biopsied Longissimus Thoracis Muscle for Estimation of Meat Quality Traits in Live Pigs
Source: Animals (Basel). 2019 Dec 19;10(1):9. doi: 10.3390/ani10010009 (PMC7022759; doi:10.3390/ani10010009)
Supplement: Supplementary file 1 [file animals-10-00009-s001.pdf]

Table S1. Summary statistics for measured traits.

Variables [1]	N	Mean	SD	Min	Max
MHC Isoforms at Biopsy					
Slow isoforms (%)	668	18.04	12.36	2.12	50.31
Fast isoforms (%)	668	81.96	12.36	49.69	97.88
Fast/Slow ratio	668	8.27	7.01	0.99	46.17
Muscle fiber characteristics at postmortem					
Total muscle fiber number ($\times 10^3$)	396	1041	246.0	420	2212
The density of muscle fibers (/mm^2)	589	254.6	52.6	123.0	521.0
Cross sectional area of muscle fiber					
Mean area	589	4095	851.4	1919	8109
Type I area	589	3326	811.4	1581	7039
Type IIA area	589	2612	756.3	567	6617
Type IIB area	589	4365	967.3	1946	8778
Proportion of muscle fiber area					
Type I	589	9.69	3.23	1.87	22.81
Type IIA	589	4.78	2.04	0.26	14.79
Type IIB	589	85.53	3.79	63.39	94.07
Proportion of muscle fiber number					
Type I	589	12.00	3.90	2.65	26.24
Type IIA	589	7.51	2.90	1.34	17.82
Type IIB	589	80.49	4.59	63.86	91.59
Meat quality					
pH_{45min}	592	6.40	0.29	5.20	7.49
L *	591	46.12	3.01	38.25	56.53
A *	591	6.61	1.16	3.46	13.41
B *	591	2.00	0.84	–0.25	5.00
FFU	569	26.41	28.50	4.00	270.0
Drip loss $_{24h}$	578	1.03	0.91	0.08	7.49
Cooking loss	595	20.08	4.64	2.81	41.94
NPPC color	580	2.52	0.65	1.00	4.50
NPPC marbling	573	1.57	0.60	1.00	6.00

[1] Legend: MHC, myosin heavy chain; FFU, filter-paper fluid uptake; NPPC: National Pork Producers Council.

Table S2. Description of cluster size by the two-step clustering analysis in pigs based on the biopsied MHC isoforms from the musculus longissimus thoracis.

		Cluster 1	Cluster 2	Cluster 3	Total
Pre-cluster	1	147 [1]	-	-	147
	2	-	-	70	70
	3	159	-	-	159
	4	-	87	-	87
	5	69	-	-	69
	6	-	82	-	82
	7	-	-	33	33
	8	4	-	-	4
	9	1	-	-	1
	10	16	-	-	16
	Total	396	169	103	668

[1] Number of individuals.

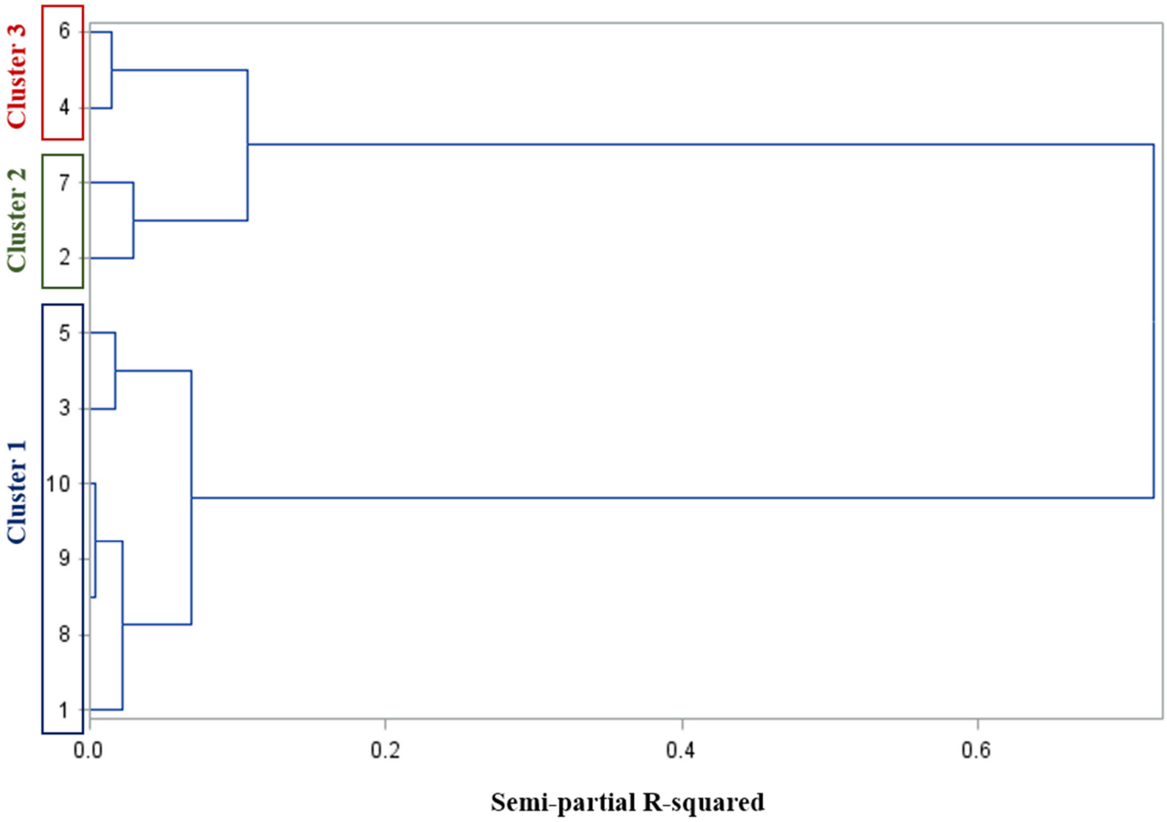

Figure S1. Cluster analysis dendrogram. For the preliminary clustering analysis, 10 pre-clusters were categorized based on the three variables of myosin heavy chain isoforms characteristics and presented by the hierarchical clustering as a tree structure [20]. Semi-partial R-squared values were used to branch distances between each level of the pre-clusters. The 10 pre-cluster groups were further categorized into three clusters using Ward's minimum-variance method [17]: blue, cluster 1 (n = 396); green, cluster 2 (n = 169); red, cluster 3 (n = 103).

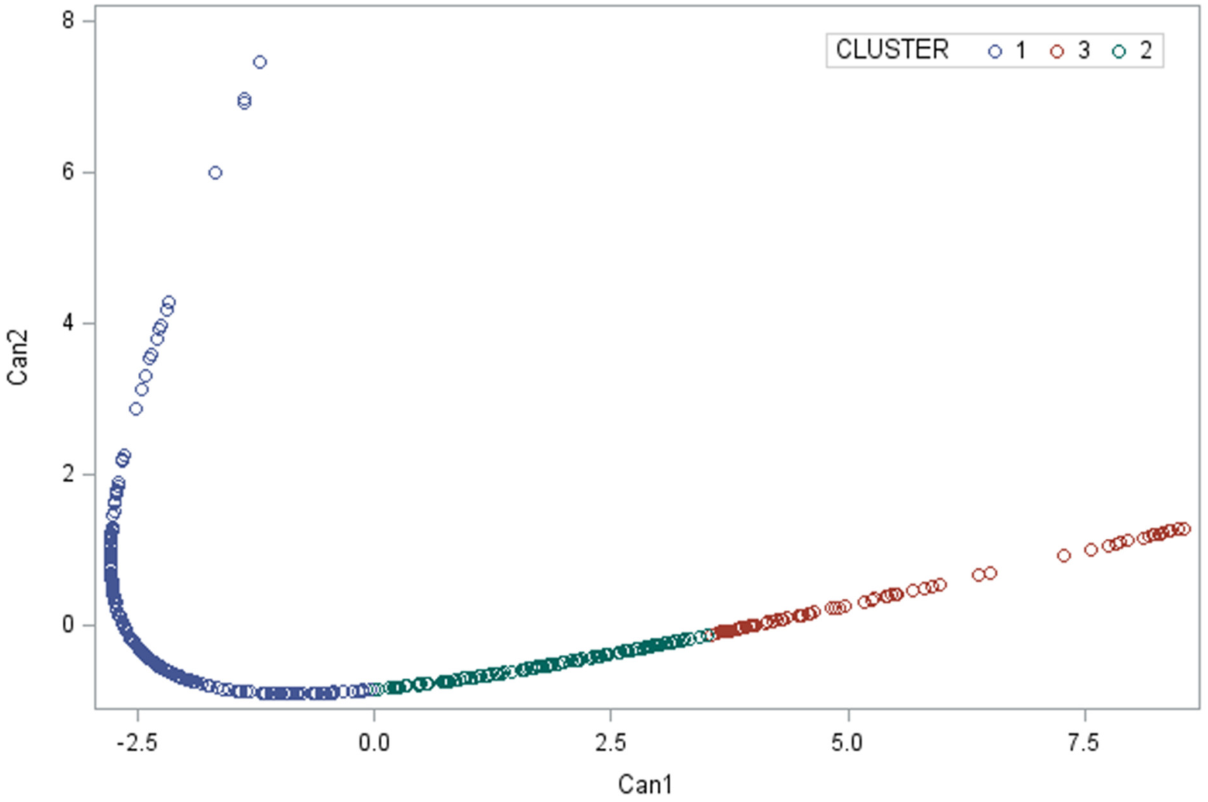

Figure S2. Grouped-scatter plot by two dimensional canonical coefficients for three clusters. The canonical coefficients were estimated by canonical discriminant analysis. The first (Can1) and second (Can2) canonical variables were used as horizontal and vertical axes, respectively. Individuals in each cluster are represented by differently colored circles: blue, cluster 1; green, cluster 2; red, cluster 3.
